# Machine learning enables prompt prediction of hydration kinetics of multicomponent cementitious systems

**DOI:** 10.1038/s41598-021-83582-6

**Published:** 2021-02-16

**Authors:** Jonathan Lapeyre, Taihao Han, Brooke Wiles, Hongyan Ma, Jie Huang, Gaurav Sant, Aditya Kumar

**Affiliations:** 1 Materials Science and Engineering, Missouri University of Science and Technology, Rolla, MO 65409 USA; 2 Civil, Architectural and Environmental Engineering, Missouri University of Science and Technology, Rolla, MO USA; 3 Electrical and Computer Engineering, Missouri University of Science and Technology, Rolla, MO USA; 4 Civil and Environmental Engineering, University of California, Los Angeles, CA USA

**Keywords:** Theory and computation, Structural materials, Composites

## Abstract

Carbonaceous (e.g., limestone) and aluminosilicate (e.g., calcined clay) mineral additives are routinely used to partially replace ordinary portland cement in concrete to alleviate its energy impact and carbon footprint. These mineral additives—depending on their physicochemical characteristics—alter the hydration behavior of cement; which, in turn, affects the evolution of microstructure of concrete, as well as the development of its properties (e.g., compressive strength). Numerical, reaction-kinetics models—e.g., phase boundary nucleation-and-growth models; which are based partly on theoretically-derived kinetic mechanisms, and partly on assumptions—are unable to produce a priori prediction of hydration kinetics of cement; especially in multicomponent systems, wherein chemical interactions among cement, water, and mineral additives occur concurrently. This paper introduces a machine learning-based methodology to enable prompt and high-fidelity prediction of time-dependent hydration kinetics of cement, both in plain and multicomponent (e.g., binary; and ternary) systems, using the system’s physicochemical characteristics as inputs. Based on a database comprising hydration kinetics profiles of 235 unique systems—encompassing 7 synthetic cements and three mineral additives with disparate physicochemical attributes—a random forests (RF) model was rigorously trained to establish the underlying composition-reactivity correlations. This training was subsequently leveraged by the RF model: to predict time-dependent hydration kinetics of cement in new, multicomponent systems; and to formulate optimal mixture designs that satisfy user-imposed kinetics criteria.

## Introduction

Concrete—a mixture of ordinary portland cement, water, and aggregates—is the foundational material used in construction of various forms of surface and sub-surface infrastructure. Much of the research in the field, in the past several years, has been focused on designing, and optimizing the performance of, blended cementitious systems, which feature 10–60%_mass_ replacement of cement with CO_2_-efficient mineral additives^1–4^; so as to offset both carbon footprint and energy impact associated with cement production and concrete design-and-use^5–10^. Examples of the aforesaid mineral additives include: (1) Fillers: limestone (crystalline calcite: CaCO_3_), and quartz (crystalline silica: SiO_2_); and (2) Pozzolans: silica fume (amorphous silicate: SiO_2_), and calcined clay such as metakaolin (dominantly amorphous Al_2_Si_2_O_7_)^11–17^. When used to partially replace cement in concrete, fillers accelerate cement hydration rates^11,12,18–20^ through provision of supplemental topographical sites for heterogenous nucleation of calcium silicate hydrate (C–S–H (In this manuscript, cement chemistry shorthand is used to represent cementitious compounds and the constituent oxides. As per this shorthand: C = CaO; A = Al_2_O_3_; S = SiO_2_; $ = SO_3_; and H = H_2_O.))—the principal *cementing* agent that binds together the particulates (e.g., of cement; aggregates; and hydration products) within the concrete microstructure. Acceleration of cement hydration, as prompted by a filler, is commonly referred to as the *filler effect*^19–21^. Along the same lines, when cement is partially replaced with a pozzolan (i.e., silicate or aluminosilicate material), the latter is known to initiate and partake in pozzolanic reaction; which involves the reaction of the pozzolan with calcium hydroxide (Ca(OH)_2_ or CH; a secondary hydration product), resulting in precipitation of additional C–S–H, thereby increasing its content in the system^18,22–24^. In addition to the aforesaid *pozzolanic effect*, pozzolans also have a finite *filler effect*; which accelerates cement hydration kinetics, especially during the first 5–0 h of hydration^18,22^.

The hydration of cement—that is, the reaction of cement with water—dictates critical physical characteristics (e.g., initial/final setting; compressive strength; chemical durability; etc.) of concrete^25–30^. Even in a plain paste—which comprises only of cement and water—hydration is a complex chemical process, with several interdependent kinetic processes (e.g., dissolution of anhydrous cement particulates; diffusion of ionic species within the microstructure; simultaneous precipitation of multiple hydration products, each dictated by a distinct mechanism; etc.) that occur concurrently. For example, the hydration of the two most reactive phases present in cement—tricalcium silicate (Ca_3_SiO_5_ or C_3_S as per cement chemistry notation); and tricalcium aluminate (Ca_3_Al_2_O_6_ or C_3_A)—are driven by disparate mechanisms; result in formation of different hydration products; and occur at distinct nonlinear, nonmonotonic rates^31^. The hydration of C_3_S results in formation of stoichiometric amounts of two hydration products (i.e., CH and C–S–H); the kinetics of C_3_S hydration is highly nonlinear with respect to time, driven dominantly by heterogeneous nucleation-and-growth of C–S–H^22,32–35^. The other cementitious phase, C_3_A, reacts vigorously upon contact with water [which, in cementitious systems, typically contains SO_4_^2−^ ions released from dissolution of C$H_2_ (gypsum); a minor phase present in cement]; as a result, small, needle-shaped crystals of ettringite (a hydration product) precipitate within minutes of mixing^36–38^. After this initial burst of precipitation, the growth of ettringite crystals occurs at a near-constant, slow rate for the ensuing hours until SO_4_^2−^ ions in the solutions are depleted^36,38,39^. In blended cementitious systems—wherein a fraction of cement is replaced with one or more mineral additives—chemical interactions among cement, water, and additives can give rise to staggeringly large degrees of freedom. This is because the nature of aforesaid interactions depends on physicochemical characteristics of the cement and the additive(s). For example, in a [cement + aluminosilicate pozzolan] system, dissolution of the pozzolan results in the release of aluminate [Al(OH)_4_^−^] and silicate [H_2_SiO_4_^2−^] ions in the contiguous solution; these ions can subsequently react with components of the paste (e.g., cement; hydration products; etc.), thereby resulting in precipitation of secondary hydration products (e.g., hydrogarnet; strätlingite; etc.) and/or stabilizing or de-stabilizing sulfate-bearing hydration products (e.g., ettringite; monosulfoaluminate; etc.)^40,41^. In [cement + aluminosilicate pozzolan + limestone] systems—apart from the aforementioned interactions—chemical reaction between the pozzolan and limestone can result in formation of additional hydration products (e.g., monocarboaluminate)^42,43^. The nature and magnitude of these effects—in addition to the above-described *filler* and *pozzolanic* effects—depend on the additives’ composition and physical characteristics (mainly specific surface area); and these effects could have profound effect on hydration kinetics of the host material, cement.

Due to the above-described complexities associated with hydration of cement in plain and multicomponent pastes, the derivation of semi-empirical equations, that describe the rate-limiting reactions or provide a plausible representation of the underlying thermo-kinetic mechanisms (e.g., equilibrium constants) that drive the reactions, is challenging^22,35,38,44–49^. Furthermore, our comprehension of how different experimental process parameters (e.g., composition of cement) affect cement hydration behavior—which, historically, have always been investigated using classical materials science approaches (e.g., the Edisonian approach of iterative synthesis-testing-analysis of specimens)—is limited, and has several knowledge-gaps. These knowledge-gaps have thus far prevented the development of all-encompassing cement hydration models capable of reliably predicting cement hydration kinetics. In spite of these knowledge-gaps, several semi-empirical numerical models (e.g., phase boundary nucleation-and-growth models) have been developed, and employed to enhance our understanding of mechanisms that drive cement hydration as function of time and various other process parameters^20,22,31,33,35,49–52^. Selected numerical models have achieved success—albeit with a caveat—in reproducing the temporal evolution of cement hydration kinetics, measured via the isothermal calorimetry technique; wherein time-dependent exothermic heat release of hydration is monitored, and used to estimate rate and extent of cement hydration^22,31–33,49^. The aforesaid caveat is that these semi-empirical models—even in the best-case scenario (e.g., after rigorous calibration of multiple parameters)—can only reproduce the results of isothermal calorimetry experiments. Simply put, they are unable to produce a priori predictions of the heat evolution profiles (or hydration kinetics profiles) of new cementitious systems. Furthermore, several researchers^31,32,49,51,53^ have argued that these models require further refinement; since models that are based on erroneous assumptions, approximations, and mechanisms, or incorrect representation of mechanisms, can still reproduce calorimetry data.

Owing to the above-described limitations of semi-empirical numerical models, in recent years, several researchers have focused on developing and applying data-driven, artificial intelligence techniques [e.g., machine learning (ML) models] to predict the properties of cementitious systems using their initial physicochemical attributes as inputs. ML models are appealing because they are able to extract composition-properties correlations in a material from the training dataset, and subsequently capitalize on such correlations to produce predictions and optimizations of properties of materials with new compositions; all without the need for an across-the-board understanding the underlying mechanisms. Even in the case of materials for which composition-properties correlations are well understood, ML models are still useful as they could be used to predict properties of materials of entirely new compositions; while being informed, guided, and constrained by well-established theoretical laws. Owing to these reasons, it is unsurprising that, just in the last ten years, numerous articles describing the use of ML models to predict properties of complex material systems (e.g., concrete; multicomponent glasses; etc.) have been published^54–65^. In our literature review, while we found scores of studies that employed ML models to predict mechanical properties of cementitious systems, we did not find any study that focused on prediction of time-dependent hydration kinetics of cement. On a much broader level, there are no studies that demonstrate the application of ML—or other analytical or statistical models, for that matter—to predict continuous, time-based evolution of material’s reactivity; although, ML models have been used to predict rate/extent of reactivity of materials (e.g., glasses) at discrete time-steps^54,59,60^. In the context of cementitious systems, prediction of continuous, time-dependent kinetics of cement hydration is valuable; because these kinetic profiles can be used to produce crude estimates of various other properties of the system^20,66–69^, such as setting time, rheological behavior, and mechanical properties (e.g., compressive strength). Simply put, time-dependent hydration kinetics of cement in a given cementitious system can be used as a singular metric to estimate—albeit roughly—various fresh and mature properties of the system. As an example: in a scenario, wherein multiple cementitious systems need to be ranked/ordered in terms of their properties (e.g., compressive strength), prompt predictions of cement hydration kinetics (produced by ML models) could be used to complement or even replace time-consuming and expensive experiments.

In this study, a random forests (RF) model^70–72^—a reformed version of the classification-and-regression decision trees (CART) model—is used to perform predictions of time-dependent, heat evolution behavior (as a measure of rate and extent of cement hydration in the first 24 h after mixing) of multicomponent cementitious systems. The RF model was trained from a high-volume database, comprising heat evolution profiles of 235 unique systems; encompassing 7 synthetic cements, and three different mineral additives (coarse limestone; fine limestone; and metakaolin) that were used—individually or in pairs—to replace 0–60%_mass_ of the cement. The aforesaid synthetic cements were formulated to encompass a wide spectrum of compositions; this was achieved by mixing three cementitious phases—C_3_S; C_3_A; and C$H_2_—at varying proportions. It should be noted that these cements represent a simplification of commercial cements, which contain two additional cementitious phases (e.g., belite or C_2_S; and ferrite or C_4_AF). These phases, nevertheless, react very slowly; and, therefore, do not significantly affect the overall hydration kinetics, at least in the first 24 h after contact with water. The RF model was rigorously trained using the database so as to establish the underlying composition-reactivity correlations. Once trained, the RF model was used to predict time-dependent hydration kinetics of cement in new, multicomponent systems. Results show that the RF model produces accurate predictions of continuous, time-dependent hydration kinetics of cement, a feat that is currently impossible with semi-empirical kinetic models. Results also show that the RF model—and the composition-reactivity correlations learned by the model during its training—can be capitalized on to predict optimal mixture designs of cementitious systems that satisfy user-imposed kinetics criteria.

## Overview of the random forests model

The random forests (RF) model—based on an ensemble of 100–1000 s of uncorrelated classification-and-regression-trees (CART)^55,58,59,73^—is used in this study. The RF model is premised on: (1) Building uncorrelated decision trees (i.e., CARTs) at a large scale; (2) Grouping CARTs into committees, so as to produce independent outputs; and (3) Averaging outputs produced by all CARTs to estimate the final output^74^. While buildings CARTs, their partitioning into terminal nodes is achieved via binary splits; the partitioning is of recursive nature, and is continued until the optimal structure of the CART is achieved (i.e., until homogeneity among terminal nodes reaches its global maximum). The RF model leverages: (1) Bagging^70,71^, which guarantees that the growth of each CART originates from a collection of bootstraps that are selected at random, with each bootstrap comprising the exact same number/type of inputs as the entire training dataset; and (2) Bootstrapping, which serves to simultaneously reduce both bias (overfitting) and variation (underfitting) amongst the large number of CARTs^75^. During the training-and-validation of the RF model, each CART is allowed to grow until it achieves its maximum-permissible size; without implementing any pre- or post-processing step (e.g., pruning; smoothening; etc.) to alter or *rectify* its structure. On account of this, the CARTs remain diverse (N.B.: Output of all CARTs are truly independent); therefore, permitting the RF model to garner input–output correlations within the training database, without disregarding data-records that digress significantly from the trends (i.e., outliers). It is worth pointing out that a pair of hyperparameters—*number of CARTs in the ensemble* and *number of logical splits in each CART*—of the RF model ought to be adjusted to achieve optimal prediction accuracy. Manual adjustment of these hyperparameters could lead to poor prediction performance. Therefore, in this study, the grid-search method^76^—coupled with the tenfold cross-validation method (see “Results and discussion” section)^58,59,77,78^—was used to autonomously adjust-and-optimize the two hyperparameters.

## Materials and methods

Tricalcium silicate (C_3_S) powder—the main precursor used for formulation of synthetic cement—was synthesized in the laboratory; details pertaining to the solid-state synthesis can be found in reference^18^. Other precursors used for formulation of synthetic cement [i.e., tricalcium aluminate (C_3_A); and gypsum (C$H_2_)], and mineral additives used to partially substitute cement [i.e., limestone (CaCO_3_); and metakaolin (Al_2_Si_2_O_7_)], were sourced, in powder form, from commercial suppliers. Reagent-grade C$H_2_ (sourced from Alfa Aesar) was determined to be 99%_mass_ pure. C_3_A (sourced from Kunshan Chinese Technology New Materials Co., Ltd) was determined to be > 98%_mass_ pure; with the remaining 2%_mass_ being free lime (CaO). Metakaolin (subsequently referred to as MK) was sourced from Imerys Kaolin; and was found to be > 98.5%_mass_ amorphous Al_2_Si_2_O_7_, with the remaining 1.5% being minor oxides of iron, calcium, titanium, and alkalis. To obtain two different particle size distributions (PSDs) of limestone (sourced from Mississippi Lime), the as-received powder (denoted as coarse limestone or C-LS) was ground using a wet, ball-milling method. Here, limestone was mixed with de-ionized water (18.3 MΩ); milled for ~ 48 h; and subsequently dried in an oven to obtain fine limestone (subsequently referred to as F-LS). PSDs of all powders were determined using a static light scattering (SLS)/laser diffraction particle size analyzer (Microtrac S3500); results are shown in Fig. S1 (Supporting Information). Details pertaining to the PSDs—including the specific surface area (SSA) of the particulates—are listed in Table S1.

7 synthetic cements—each with a distinct composition—were formulated by mixing C_3_S, C_3_A, and C$H_2_ at varying mixture proportions (see Table 1). Emphasis was given to vary the C_3_S-to-C_3_A and C_3_A-to-C$H_2_ ratios so as to emulate the compositions of commercial cements, which feature broad-range variations in C_3_S, C_3_A, and C$H_2_ contents. Next, pastes were formulated by mixing the solids (i.e., cement; or cement + mineral additives) with DI-Water (MΩ 18.3) at a constant liquid-to-solid mass ratio (*l/s*) of 0.45. Plain pastes were formulated by mixing cement with water. Binary pastes were formulated by partially replacing the cement with an additive (either coarse limestone; or fine limestone; or metakaolin) at replacement levels of 10–60%_mass_. For ternary pastes, up to 60%_mass_ of cement was replaced with a [metakaolin + limestone] mixture, prepared at mass ratios of either 1:1 or 2:1 (between masses of metakaolin and limestone). Mixture designs, featuring binary and ternary pastes prepared using Cement #1, are shown in Table 2. Binary and ternary pastes, prepared using Cements #2–#7, were formulated using the same designs of mixtures shown in Table 2.

**Table 1:** Compositions of cements #1–7.

Cement no. (#)	C_3_S (%_Mass_)	C_3_A (%_mass_)	C$H_2_ (%_mass_)	C$H_2_/C_3_A (mass ratio)
1	90	4	6	1.50
2	92	4	4	1.00
3	88	8	4	0.50
4	80	8	12	1.50
5	70	12	18	1.50
6	82	12	6	0.50
7	100	0	0	N/A

As can be seen, the synthetic cements feature a wide range of compositions, with different C_3_S, C_3_A, and C$H_2_ contents.

**Table 2:** Mixture designs of binary and ternary pastes prepared using Cement #1.

C_3_S (%_mass_)	C_3_A (%_mass_)	C$H_2_ (%_mass_)	Cement #1 (%_mass_)	MK (%_mass_)	LS (%_mass_)	Total additive (%_mass_)
81	3.6	5.4	90	10	0	10
72	3.2	4.8	80	20	0	20
63	2.8	4.2	70	30	0	30
54	2.4	3.6	60	40	0	40
45	2	3.0	50	50	0	50
36	1.6	2.4	40	60	0	60
72	3.2	4.8	80	10	10	20
63	2.8	4.2	70	15	15	30
54	2.4	3.6	60	20	20	40
45	2.0	3.0	50	25	25	50
36	1.6	2.4	40	30	30	60
63	2.8	4.2	70	20	10	30
36	1.6	2.4	40	40	20	60

The ternary paste features either a 1:1 or a 2:1 ratio between metakaolin (MK) and limestone (LS). Limestone with two different particle size distributions—i.e., coarse limestone (C-LS) and fine limestone (F-LS)—are used.

Hydration kinetics of cement in the pastes were measured using a TAM IV (TA Instruments) isothermal conduction microcalorimeter over a 24-h period at a constant temperature of 20 ± 0.1 °C. The microcalorimeter monitors and records the rate (mW. g_Cem_^−1^) and extent (J. g_Cem_^−1^) of heat released from the hydration of cement in the pastes at a high time-resolution of 1 μs and power-resolution of 0.1 μW. The heat flow rate and cumulative heat release serve as direct, accurate measurements of rate (kinetics) and degree (extent) of hydration of cement, respectively^18,22,23,49,79^.

## Database and appraisal of prediction accuracy of the random forests model

Heat evolution (calorimetry) profiles obtained from microcalorimetry experiments—that is, time-dependent heat flow rate and cumulative heat release corresponding to hydration of cement in the pastes—were compiled, and split into two databases: a training database (Table S2; Supporting Information) comprising 5640 data-records from 235 pastes (including 18 pastes that were repeated to account for standard deviation in isothermal microcalorimetry profiles); and a testing database (Table S3) consisting of 168 data-records from 14 unique pastes. It should be noted that while heat flow rate and cumulative heat release profiles correspond to differential and cumulative forms of heat release behavior, in this study, both profiles were treated (i.e., for training and testing) separately. This was done to evaluate the prediction performance of the ML model on two separate but interdependent measures of reactivity, wherein the differential profile is comparatively more complex (highly nonmonotonic due to its transition through different stages such as dissolution and induction period) and more sensitive to minor changes in physicochemical attributes of the paste.

The training database was used to train the RF model and optimize its hyperparameters. Next, the model’s prediction accuracy—that is, its ability to produce a priori predictions of time-dependent heat flow rate and cumulative heat release of pastes with new compositions—was evaluated against pastes included in the testing database. Both the training and testing databases included pastes formulated using each of 7 synthetic cements, and each of three additives [i.e., metakaolin (MK); coarse limestone (C-LS); and fine limestone (F-LS)]. Both databases included 7 inputs pertaining to physicochemical properties of the paste: C_3_S content (%_mass_); C_3_A content (%_mass_); C$H_2_ content (%_mass_); metakaolin content (%_mass_); limestone content (%_mass_); SSA of limestone particulates (cm^2^. g^−1^); and time (hour). The outputs comprised of time-dependent cumulative heat release (J. g_Cem_^−1^) and heat flow rate (mW. g_Cem_^−1^) from 0-to-24 h, with a 1-h time-interval between successive steps. Statistical parameters pertaining to the training and testing databases are summarized in Tables S2 and S3, respectively.

To evaluate the prediction performance of the RF model, predictions of heat flow rate and cumulative heat release of pastes in the testing database were compared against isothermal calorimetry measurements. These predictions of heat release profiles were produced by the model with a 2-h interval. Here, a time-step of 2 h was chosen so as to have a discernible gap between successive predictions that can be easily visualized. It is worth clarifying that predictions could have been produced with a time-interval of 1 h (or 0.5 h or 3 h); but doing so would not change the overall prediction performance of the model. Five unique statistical parameters were used to quantitatively compare the predictions against the measurements. The five statistical parameters include: mean absolute percentage error (MAPE); Person correlation coefficient (R); mean absolute error (MAE); coefficient of determination (R^2^); and root mean squared error (RMSE). Each parameter serves as a distinct measure of prediction error; and equations describing how these parameters are estimated are described in prior studies^55,59^.

## Results and discussion

### Isothermal microcalorimetry: hydration kinetics of cement in multicomponent pastes

**Figure 1:**
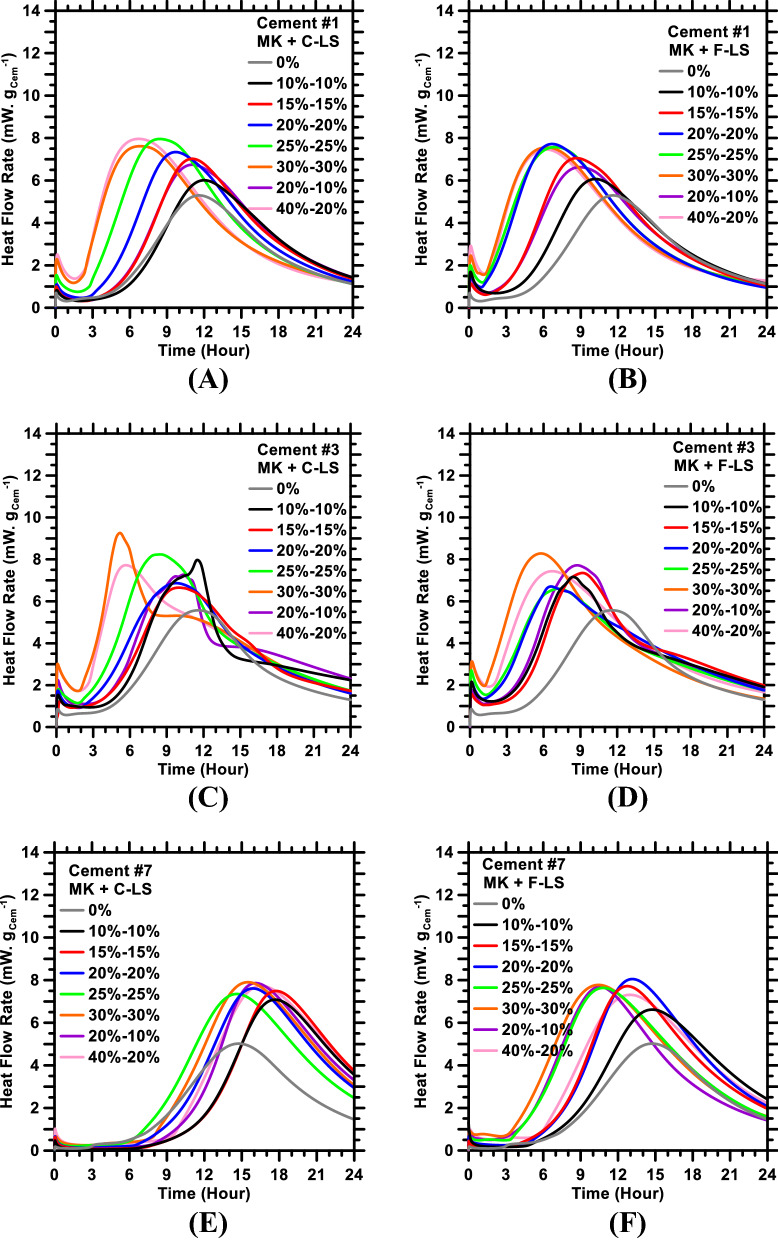
Heat flow rate profiles of ternary pastes prepared using Cement #: (**A**)1; (**C**) 3; and (**E**) 7, wherein the cement is partially replaced with metakaolin (MK) and coarse limestone (C-LS). Heat flow rate profiles of pastes containing fine limestone (F-LS) and metakaolin are shown in (**B**, **D**, and **F**)_**.**_ The replacement levels of cement with metakaolin and limestone are shown in the legends (e.g., 40%-20% indicates 40%_mass_ and 20%_mass_ replacement of cement with metakaolin and limestone, respectively).

Figure 1 shows representative hydration kinetics profiles of cement (i.e., time-dependent heat flow rate profiles) in ternary pastes formulated using three different synthetic cements (i.e., Cement #1; #3; and #7) and three different mineral additives (i.e., metakaolin; coarse limestone; and fine limestone) at 0–60%_mass_ cement replacement levels. As can be seen, with increasing levels of replacement of cement with additives, there is: either (1) A leftward shift in main peak of the heat flow rate profile, which is indicative of enhancement in cement hydration rates^19,20,22,80^; or (2) A rightward shift, which is indicative of suppression of cement hydration kinetics^22,49,67,79,81–83^. The magnitudes of these alterations—that is, acceleration or deceleration of cement hydration kinetics—strongly depend on multiple factors: cement’s composition; PSD and composition of the additive; and overall mixture design (i.e., %_mass_ of cement; limestone; and metakaolin in paste). For example, in pastes formulated using Cement #1 (Fig. 1A,B), cement hydration rates are enhanced—in near-monotonic manner—with increasing levels of cement-replacement with additives. Notwithstanding, depending on the PSD of limestone (i.e., coarse limestone; or fine limestone) and the overall mixture design, the magnitudes of these enhancements are different from each other. In pastes formulated using Cement #3 (Fig. 1C,D)—on account of disparate, albeit concurrent, physical/chemical interactions among components of the paste—the heat flow rate profiles feature multiple peaks. Here, the physical interactions may include filler effects induced by metakaolin and limestone, resulting in enhancement of hydration kinetics of C_3_S. The chemical interactions may include those among metakaolin, limestone, C_3_A, C$H_2_—resulting in alterations in the stability of SO_3_-bearing phases (e.g., ettringite), and precipitation of CO_3_-bearing phases (e.g., monocarboaluminate)^42,43,84^. Lastly, in pastes formulated using Cement #7 (Fig. 1E), in which the cement is partially replaced with metakaolin and coarse limestone, the main hydration peak appears to shift rightward—as opposed to the leftward shift observed in other pastes—in relation to increasing levels of replacement of cement with additives. As Cement #7 is comprised of only C_3_S, it is expected that aluminate anions [Al(OH)_4_^¯^], released from metakaolin’s dissolution upon contact with water, inhibit topographical sites (e.g., etch-pits; grain boundaries; etc.) of C_3_S dissolution and of C–S–H’s nucleation-and-growth; thereby resulting in slower hydration kinetics of C_3_S (and thus of cement)^18,85–87^. However, when fine limestone is used to partially replace cement, the metakaolin-induced inhibition of early age hydration of C_3_S appears to be less pronounced (Fig. 1F); conceivably due to the acceleration induced by *filler effect* of the fine limestone.

**Figure 2:**
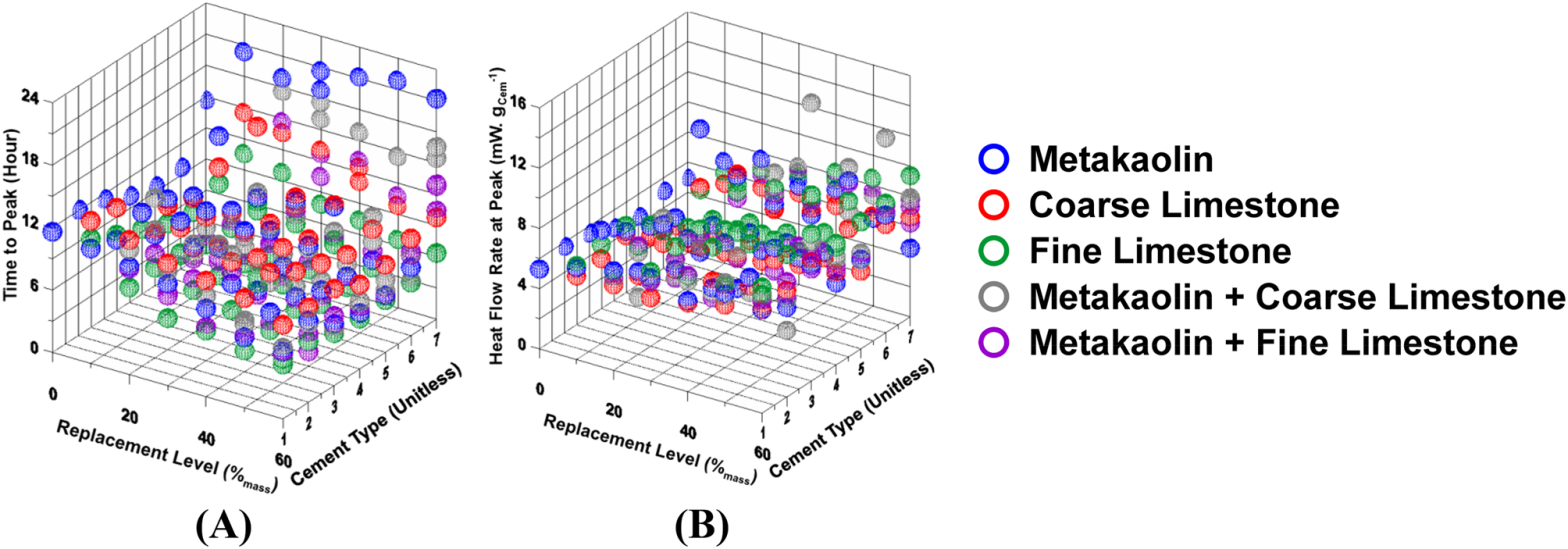
Characteristic calorimetric (kinetic) parameters of all pastes, prepared using 7 different synthetic cements, plotted as functions of the replacement level (%_mass_) of cement with different additives (i.e., metakaolin; coarse limestone; and fine limestone). Representative calorimetric parameters shown here include: (**A**) Time to main heat flow rate (or hydration) peak; and (**B**) heat flow rate at the peak.

Figure 2—which shows two characteristic kinetic parameters (i.e., intensity of heat flow rate at the main hydration peak, and time to main hydration peak; extracted from the heat flow rate profiles^19,20,22,80^) of pastes—highlights the complex nature of composition-reactivity correlations in multicomponent pastes. As can be seen, depending on initial physicochemical properties of the paste (i.e., cement composition; cement replacement level; composition/PSD of the additives; and mixture design), the kinetic parameters change drastically (e.g., time to peak ranges from 4.5-to-22 h). Such complexity, nevertheless, is not unforeseen; and can be attributed to the ability of each physicochemical parameter—e.g., cement composition; additive’s chemical makeup; etc.—to cast distinct, and substantial, effect on the hydration kinetics of one or both cementitious phases (i.e., C_3_S and C_3_A), and thus of cement. Going from one paste to another, when multiple physicochemical parameters are concurrently varied, the net effect on cement hydration kinetics is the culmination of not only the sum of individual contributions of each parameter, but also mutual interactions between the said parameters. While decades of research on understanding the mechanisms that drive cement hydration has collectively provided valuable insights, and led to the development of semi-empirical numerical models; there still are significant knowledge-gaps that disallow a priori prediction of time-dependent cement hydration behavior, especially in multicomponent pastes (e.g., pastes shown in Fig. 2; which feature complex, highly nonlinear linkages between reactivity and composition of paste components). Explicitly because of this reason, data-driven methods—that do not require an across-the-board understanding the underlying mechanisms—are needed for such predictions.

### Prediction of time-dependent hydration kinetics of cement

**Figure 3:**
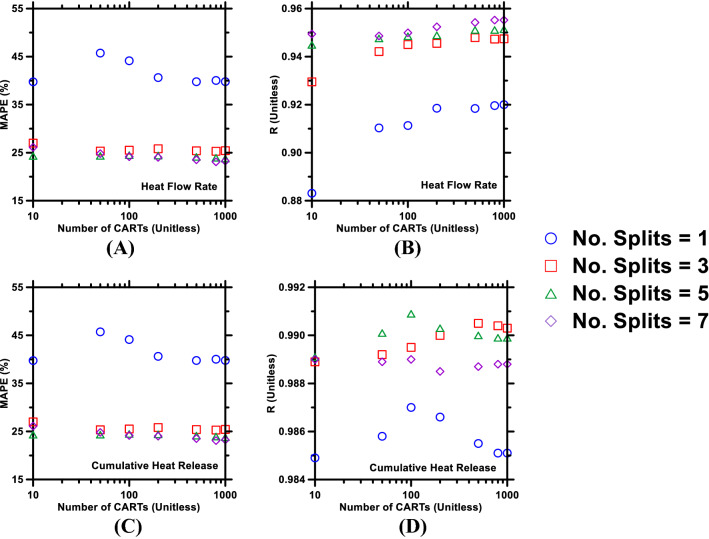
Grid-search method used to optimize hyperparameters (*number of CARTs in the ensemble*; and *number of logical splits at each node of the CART*) of the RF model to improve its prediction performance against: heat flow rate, assessed using (**A**) MAPE, and (**B**) R; and cumulative heat release, assessed using (**C**) MAPE, and (**D**) R.

It is worth reiterating here that, in this study, the training of the RF model was accomplished using a fraction of the database (i.e., training database); and, post-training, the model’s prediction performance was ascertained by comparing its predictions of time-dependent heat evolution profiles of pastes (included in the testing database) against experimentally measured profiles. In general, to maximize the RF model’s ability to perform predictions over blank data-domains, it is crucial that all of the following criteria are met: the model is offered adequate training so as to develop logical correlations between inputs (e.g., cement composition; mixture design; etc.) and outputs (i.e., time-dependent heat flow rate and cumulative heat release); Outliers in the database are incorporated rather than discarded (e.g., pruned from the CARTs); and Both underfitting and overfitting (i.e., variance and bias among CARTs) are abated inasmuch as they can be. Towards fulfilling the aforementioned criteria, the pair of *free* hyperparameters of the model (no. of CARTs in the ensemble; and no. of logical splits in each CART) were autonomously adjusted-and-optimized using the grid-search method^76,88^. The grid-search method—which is employed in tandem with the tenfold cross-validation method^78^—performs by grid-by-grid search of optimal magnitudes of the hyperparameters such that the model’s predictions digress from actual observations (measured using the five statistical parameters listed in “Database and appraisal of prediction accuracy of the random forests model” section) by as little as possible. Representative results, obtained from the grid-search method, are shown in Fig. 3. Here, two statistical parameters (i.e., R; and MAPE) are used as archetypal metrics to measure the average deviation between predictions and measured values of cumulative heat release and heat flow rate over a 24-h period. Based on these metrics, it was found that, for predictions of cumulative heat release, the optimal values of *no. of CARTs (trees) in the ensemble (forest)* and *no. of logical splits in each CART* were found to be 500 and 5, respectively. For predictions of heat flow rate, the optimal values were slightly higher—that is, 800 and 7, respectively—presumably because of highly nonlinear and nonmonotonic nature of heat flow rate with respect to time [N.B.: Cumulative heat release increases monotonically—although in nonlinear fashion—with respect to time]. The grid-search method revealed that when the *no. of logical splits per CART* were: (1) Less than the optimal value, the decision-trees were not adequately sophisticated to process the complex, inputs-outputs correlations from the root node to the terminal, leaf nodes; and (2) Larger than the optimal value, the root-to-leaf structure of the CARTs became too complex, thus amplifying bias (or the likelihood of overfitting, wherein outliers in the database are prioritized to similar degree as data-records that fall into trends). Similarly, when the *number of CARTs in the ensemble* was: (1) Less than the optimal number, the shortage of independent bootstraps prevented the model from instituting reliable inputs-outputs correlations from the training database; and (2) Larger than the optimal number, a large numbers of CARTs ended up having the same structure, thus resulting in redundancy and little-to-no improvement in the model’s overall prediction performance in spite of the undesired increase in computational complexity^70,89^.

**Figure 4:**
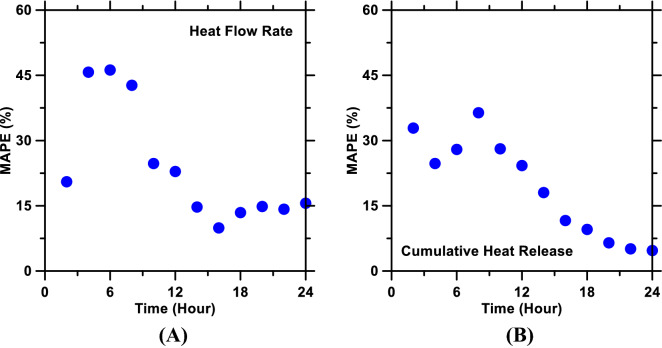
Mean absolute percentage error (MAPE)—as a measure of error in prediction (with respect to measurements)—of (**A**) heat flow rate and (**B**) cumulative heat release of pastes over a 24-h period. MAPE was estimated at different time intervals; as such, the plots show MAPE as functions of time.

**Figure 5:**
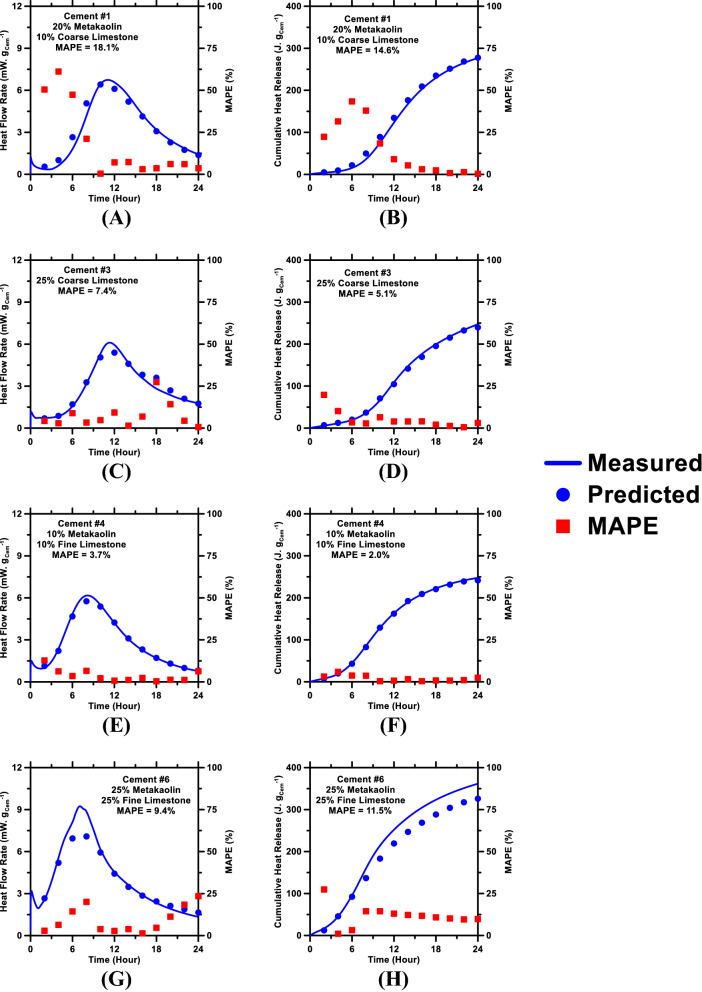
The RF model’s predictions of: (**A**) heat flow rate and (**B**) cumulative heat release of Cement #1 + metakaolin + coarse limestone paste; (**C**) heat flow rate and (**D**) cumulative heat release of Cement #3 + coarse limestone paste; (**E**) heat flow rate and (**F**) cumulative heat release of Cement #4 + metakaolin + fine limestone paste; and (**G**) heat flow rate and (**H**) cumulative heat release of Cement #6 + metakaolin + fine limestone paste compared against experimental measurements. Mean absolute percentage error (MAPE) as function of time are shown in each figure. The time-averaged MAPE of the predictions are shown in the legends.

Following the optimization of the RF model’s hyperparameters, the model was used to predict time-dependent heat flow rate and cumulative heat release profiles—as proxies of cement hydration kinetics—of pastes included in the testing database (N. B.: The RF model, during its training, was not exposed to data pertaining to these pastes). Deviations between predictions and measured values of heat flow rate and cumulative heat release, as functions of time, were quantified using the five statistical parameters described in “Database and appraisal of prediction accuracy of the random forests model” section. These parameters—pertaining to prediction errors of heat flow rate and cumulative heat release—are shown in Figs. S2 and S3 (Supporting Information), respectively; representative results [MAPE of prediction errors in relation to time] are shown in Fig. 4. These prediction errors were then averaged over the 24-h period; these time-averaged errors are summarized in Table 3. Figure 5 shows predictions of heat evolution profiles of representative pastes (belonging to the testing database) plotted against those measured using isothermal microcalorimetry. Figure S4 (Supporting Information) shows the learning behavior of model; specifically, the enhancement in the prediction performance of the RF model (with optimized parameters) in relation to increasing volume of the training database. Figure S5, shows the variable importance analysis; specifically, the ranking of physicochemical attributes of the pastes in terms of the influence they exert on the pastes’ heat release behavior. Discussion pertaining to results shown in Figs. S4 and S5 is provided in the Supporting Information.

**Table 3:** Time-averaged magnitudes of statistical parameters, used to quantify errors in RF model’s predictions of cumulative heat release and heat flow rate.

ML model	R	R^2^	MAE	MAPE	RMSE
	Unitless	Unitless	mW. g_Cem_^−1^	%	mW. g_Cem_^−1^
**Heat flow rate**					
RF	0.952	0.912	0.410	23.09	0.63

	Unitless	Unitless	J. g_Cem_^−1^	%	J. g_Cem_^−1^
**Cumulative heat release**					
RF	0.991	0.982	10.45	19.15	14.90

As can be seen in Figs. 4, 5 and Table 3, heat flow rate and cumulative heat release profiles of the pastes, as predicted by the RF model, are in excellent agreement with experimental measurements. R^2^ and RMSE of heat flow rate predictions are 0.91 and 0.63 mW. g_Cem_^−1^, respectively; while the R^2^ and RMSE of cumulative heat release predictions are 0.98 and 14.9 J. g_Cem_^−1^, respectively. These results reinforce the point made earlier in “Introduction” section—that data-driven, artificial intelligence-based models are able to predict outcomes of new systems, with much higher accuracy as compared to semi-empirical models, while obviating the need for thorough comprehension of the mechanisms that dictate cause-effect correlations in such systems. Statistical parameters shown in Figs. 4, S2, and S3 reveal an important trend: the RF model produces more accurate predictions of cement hydration kinetics at later ages (i.e., time ≳ 5 h) as compared to predictions of early age (time ≲ 5 h) hydration kinetics. This inconsistency—in prediction accuracy of the model at early versus later ages—is hypothesized to be caused by the inherent, substantial differences in the complexity of early vis-à-vis later age hydration kinetics of cement. More specifically, cement hydration transitions through four different stages—*initial dissolution period*; *induction period*; and *acceleration period*—within the first 2–5 h; and each stage leaves a unique *footprint* on the hydration kinetics (and, therefore, heat evolution) profile owing to the disparities in the underlying mechanisms^31,32^. However, between 5-and-24 h of hydration (and even beyond 24 h), cement hydration transitions through only two stages (i.e., *acceleration period*; and *deceleration period*); and, more importantly, during both stages, cement hydration kinetics are driven by a singular, nucleation-and-growth mechanism^22,32,33,49,81^. Furthermore, early age hydration kinetics of cement are more susceptible to changes (i.e., acceleration or deceleration) in response to even minor alterations in the system’s physicochemical parameters (e.g., changes in composition/content of cement; composition/PSD/content of additive; etc.). Therefore, it is conceivable that the RF model would require a very large volume of database, with significant diversity, to capture such dynamic and rapid variations in early age hydration kinetics of cement induced by various physicochemical factors (see Figure S4). Later age hydration kinetics of cement—in contrast to early age—are appreciably less sensitive to the aforementioned physicochemical factors; and, therefore, can be predicted with higher accuracy.

Comparisons of predicted kinetics profiles with measured ones (Fig. 5G,H) also revealed another important point. Predictions of cement hydration kinetics in metakaolin-rich pastes were, in general, found to be less accurate compared to all other pastes. This discrepancy is expected to be a manifestation of the ability of metakaolin to affect cement hydration kinetics—especially at early ages—in ways that are substantially different from those induced by limestone (or other fillers, for that matter). As described in “Introduction” section, in metakaolin-rich systems, the dissolution of metakaolin releases aluminate ions [Al(OH)_4_^−^] into the contiguous solution; these ions inhibit C_3_S dissolution sites, as well as heterogeneous nucleation sites for C–S–H, thus resulting in suppression of early age hydration of C_3_S^18,22,23,80^. Furthermore, metakaolin exerts dual effects—filler and pozzolanic effects—of which the former accelerates early age hydration kinetics of C_3_S^18,23^. Lastly, in ternary pastes, interactions among C_3_A, metakaolin, limestone, and C$H_2_ can result in formation of CO_3_-bearing phases (e.g., monocarboaluminate) at the expense of SO_3_-bearing monosulfoaluminate phase; thereby affecting the overall hydration behavior of cement^5,90^. As would be expected, the nature and magnitude of these effects depend strongly on cement chemistry (e.g., %_mass_ of C_3_A and C$H_2_ in the cement). These effects are absent or negligible in metakaolin-deficient pastes; or in pastes formulated using small amount of metakaolin and large amount of fine limestone, wherein the latter acts to accelerate C_3_S hydration kinetics, thereby counteracting the hydration-inhibiting effects of metakaolin. It is expected that supplementing the training database with more data-records of metakaolin-rich systems can allow the RF model to better grasp the effects of metakaolin; thereby allowing it to produce more accurate predictions of cement hydration kinetics in binary and ternary metakaolin-rich pastes.

The proficiency of the RF model’s in terms of promptly producing high-fidelity predictions of cement hydration kinetics (in the forms of time-dependent cumulative heat release and heat flow rate) is anticipated, given the large body of published research^54–56,58,59,73,77,91^; in which it has consistently been shown that the RF model—and other decision trees-based models—generally outperform other models that are based on nonlinear regression analyses (e.g., elastic net regression), or an assortment of logistic transfer functions (e.g., artificial neural networks), or data clustering/mapping mechanisms (e.g., support vector machines). To benchmark the prediction performance of the RF model, two additional ML models developed and used in our previous studies^55,57–59,92^—the multilayer perceptron artificial neural network (MLP-ANN) model; and support vector machine (SVM)—were used to produce predictions of heat release behavior, and compared against those produced by the RF model (see Fig. S6; Supporting Information). As shown in Figure S6, the RF model consistently produces more accurate predictions that the other two ML models. The RF model’s superior prediction performance can be attributed to aspects of its internal architecture^70,71,93^; the important ones of which are summarized ahead. In conventional decision trees-based models, a large number of CARTs are grown with minimal regularization; because of this, the accuracy of the model’s predictions is predicated on the database’s diversity and its volume being large (e.g., >  > 1000 unique data-records). In the RF model, used in this study, this deficiency (requirement of high-volume database) was overcome by introducing regularization for each CART through several mechanisms: (1) Minimizing variance (underfitting): through construction of an enormous number (≫ 100) of deep, unpruned CARTs, on a node-by-node basis; (2) Establishing robust cause-effect correlations within the training database: by allowing unmitigated growth of each CART until it reaches its maximum-allowable size, while revoking the criterion that the number of terminal (leaf) nodes among different CARTs ought to be the same; (3) Minimizing bias (overfitting): by adopting 3-stage randomization, which ensures that each CART—of the 100 s that are included in the ensemble—produces its own, unique prediction that is truly independent of all other CARTs; and (4) Autonomous optimization: by utilizing the tenfold cross-validation method^78^ in tandem with the grid-search method^88,91^ to autonomously adjust-and-optimize the pair of hyperparameters (i.e., number and structure of CARTs) so as to establish robust inputs-outputs correlations while also accounting for outliers in the database.

### Optimization of mixture design to meet target kinetics criteria

Results shown in “Prediction of time-dependent hydration kinetics of cement” section prove that the RF model—provided that is rigorously trained, and its hyperparameters are optimized—can be utilized to promptly and reliably predict the time-dependent heat flow rate and cumulative heat release corresponding to hydration of cement in multicomponent systems. The authors posit that this ability of the RF model—to understand hidden correlations between physiochemical attributes of pastes and hydration behavior of cement in such pastes—can be leveraged to develop optimal mixture design of [cement + mineral additive] systems that exhibit target (user-imposed) hydration behavior. To verify this, a Bayesian optimization module^58,59,91,94,95^ was designed—atop the *prediction module* of the RF model—to enable predictions of optimal mixture design of multicomponent [cement + metakaolin + limestone] pastes that would satisfy target kinetics criteria (i.e., a target cumulative heat value at 24 h).

**Figure 6:**
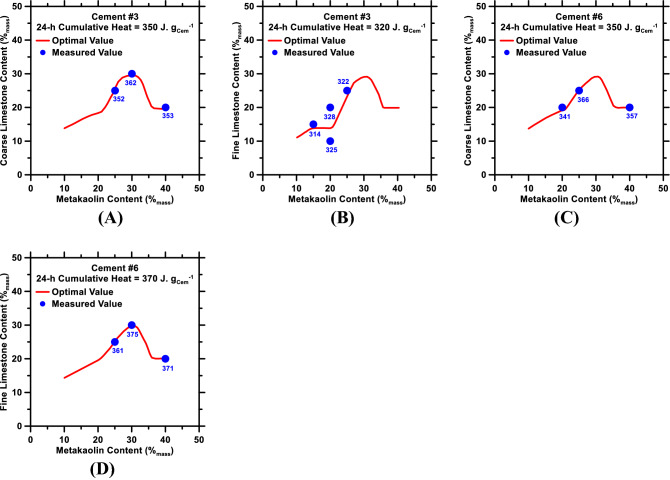
Results produced by the RF-based optimization module. Optimum levels (%_mass_) of replacement of cement with metakaolin (x-axis) and limestone (y-axis) in ternary pastes formulated using: (**A**) Cement #3 + metakaolin + coarse limestone; (**B**) Cement #3 + metakaolin + fine limestone; (**C**) Cement #6 + metakaolin + coarse limestone; (**D**) Cement #6 + metakaolin + fine limestone. The target 24-h cumulative heat release is indicated in legend of each figure. Data-points (solid-blue symbols) from experimental measurements—and their corresponding 24-h cumulative heat release values—are also included in each figure.

Within the optimization scheme, the target 24-h cumulative heat release was set at 320–370 J. g_Cem_^−1^—which are reasonably high values that entail high degree/extent of cement hydration (i.e., ~ 67–77%, assuming the enthalpy of cement hydration is 480 J. g_Cem_^−1^). Next, two mixtures—[(100 − *x* − *y*) %_mass_ Cement #3 + *x* %_mass_ metakaolin + *y* %_mass_ limestone] and [(100 − *x* − *y*) %_mass_ Cement #6 + *x* %_mass_ metakaolin + *y* %_mass_ limestone]—were designed. And, in the final step, the RF-based optimization module was implemented to estimate the optimum levels of replacement of cement with metakaolin (*x*) and (fine or coarse) limestone (*y*) that would result in the target 24-h cumulative heat release. Results produced by the optimization module are shown in Fig. 6. For the purposes of benchmarking the optimization results, data-points obtained from isothermal microcalorimetry experiments of ternary pastes—in which the 24-h cumulative heat release is within ± 5% of the target value—are also included in Fig. 6.

As shown in Fig. 6, results produced by the RF-based optimization module are in good agreement with data-points extracted from isothermal microcalorimetry experiments. While this agreement between predictions and experiments is reassuring, the trend(s) that surfaced in Fig. 6A–D are counterintuitive. For example, in the figures, there is a common trend that emerged: In order to achieve a high degree/extent of cement hydration, the limestone content needs to be increased with increasing metakaolin content; that is, from 10-to-30%_mass_ in the paste. While this correlation between limestone and metakaolin contents cannot be fully justified from theory, it is expected that higher limestone content—and its associated filler effect—is needed to overcome metakaolin’s propensity to suppress hydration of the C_3_S phase in cement. Higher limestone content would also be needed to chemically interact with metakaolin to produce CO_3_-bearing phases (e.g., monocarboaluminate); thereby resulting in high overall reactivity of cement at 24 h. When the metakaolin content is very high (i.e., ≳ 30%_mass_), the optimization results (Fig. 6) suggest that the limestone content ought to be reduced to ~ 20%_mass_ to achieve high degree of cement hydration. Again, the underlying reasons for this are unclear; but it is hypothesized that large content of metakaolin in the paste ensures elevated filler and pozzolanic (i.e., reaction between metakaolin and calcium hydroxide) effects of metakaolin, thereby requiring less limestone to achieve high degree of cement hydration. Large content of metakaolin in the paste could also result in chemical reaction with CSH_2_, leading to formation of SO_3_-bearing ettringite and monosulfoaluminate phases; thus, precluding the need for large content of limestone.

The discussion above suggests that results produced by the RF-based optimization module cannot be justified by theory—at least not in a comprehensive manner. Nonetheless, the excellent agreement between experiments and optimization results (Fig. 6) supports the premise that the optimization module can produce reliable results even when the underlying mechanisms are not well understood. This is an important point; the implication of which is that the RF model, once trained with a database with additional data-entries, could be employed to promptly and reliably predict the ideal amount and type of additive(s) for a [cement + mineral additive(s)] system that exhibits desired time-dependent heat evolution profile, while concurrently fulfilling desired performance (e.g., desired setting time; and desired 28-day compressive strength) and sustainability (e.g., cement content < 50%_mass_ of the binder) criteria.

## Conclusions

Concrete—the most produced-and-used material in the world—is the foundational material used in construction of various forms of infrastructure. Much of the research in the past several years has been focused on designing, and optimizing the performance of, blended cementitious systems, which feature 10–60%_mass_ of cement replaced with CO_2_-efficient mineral additives; so as to offset both carbon footprint and energy impact associated with cement and concrete production. The hydration of cement—a complex chemical phenomenon comprising multiple, concurrent dissolution–precipitation processes—dictates critical physical characteristics (e.g., initial/final setting; compressive strength; chemical durability; etc.) of all cement-based systems (e.g., concrete). Therefore, prediction of continuous, time-dependent kinetics of cement hydration is valuable; because these kinetic profiles could be used as a singular metric to estimate various fresh and mature physical properties of the host material. Notwithstanding, semi-empirical kinetics models (e.g., phase boundary nucleation-and-growth models)—which are based partly on theoretically-derived kinetic mechanisms, and partly on assumptions (to fulfill knowledge-gaps)—are unable to produce a priori prediction of hydration kinetics of cement; especially in multicomponent systems, wherein chemical interactions among the system’s components occur concurrently, thus giving rise to a staggeringly large degrees of freedom.

The work presented in this paper is a pioneering effort to enable prompt, high-fidelity, and a priori predictions of continuous, time-dependent hydration kinetics of cement in multicomponent systems (i.e., plain; binary; and ternary pastes). To the best of authors’ knowledge, there are no studies that have demonstrated the use of machine learning—or other sophisticated statistical models, for that matter—to predict continuous, time-resolved evolution of reactivity of multiphase material such as cement. In this study, a modified form of the random forests (RF) model was developed; and subsequently trained from a high-volume database. The database comprised of time-resolved kinetics profiles of 100 s of unique systems encompassing 7 synthetic cements, and three different mineral additives (i.e., limestone with two different PSDs; and metakaolin) that were used—individually or in pairs—to replace 0–60%_mass_ of the cement. The aforesaid synthetic cements were formulated to encompass a wide spectrum of compositions; this was achieved by mixing three cementitious phases—C_3_S; C_3_A; and C$H_2_—at varying proportions. Results show that the RF model—once trained, and then fed with information (as inputs) pertaining to physicochemical characteristics of multicomponent pastes—produces accurate, a priori predictions of continuous, time-dependent hydration kinetics of cement in multicomponent pastes; a feat that is currently impossible with semi-empirical kinetic models. Results also show that the RF model—and the composition-reactivity correlations learned by the model during its training—can be capitalized on, and coupled with Bayesian optimization scheme, to predict optimal mixture designs of cementitious systems that satisfy user-imposed kinetics criteria.

This study demonstrates the use of a machine learning model—trained-and-validated from rigorous experimentation—to enable high-fidelity prediction of cement hydration behavior in multicomponent systems, as well as to formulate mixture designs that satisfy target (user-imposed) kinetic criteria; all without a comprehensive understanding of the underlying kinetic mechanisms. In the context of optimization, the machine learning model—once trained with a database with additional data-entries—could be employed to predict the ideal amount and type of additive(s) for a [cement + mineral additive(s)] system that exhibits desired time-dependent cement hydration kinetics, while concurrently fulfilling desired performance (e.g., desired setting time; and desired 28-day compressive strength) and sustainability (e.g., cement content < 50%_mass_) criteria. Furthermore, the machine learning methodology described in this study is generic; in that it can be readily extended to describe time-dependent dissolution–precipitation interactions in other material systems (e.g., dissolution of glass in solvent; time-dependent degradation of material in relation to environmental variables; etc.). In summary, it can be said that the novel work presented in this study represents an important starting point for artificial intelligence-based mixture design optimization of complex material systems (e.g., cementitious materials)—which is in line with the “materials-by-design” paradigm—by leveraging the knowledge of the systems’ underlying nonlinear composition-property (i.e., reactivity) relationships.

## Supplementary Information

Supplementary Information

## Data Availability

The datasets presented herein, the machine learning model, and the code generated or used during the study are available from the corresponding author (A. Kumar; kumarad@mst.edu) by request.
