# Supplementary material for: Machine learning enables prompt prediction of hydration kinetics of multicomponent cementitious systems
Source: Sci Rep. 2021 Feb 16;11:3922. doi: 10.1038/s41598-021-83582-6 (PMC7886887; doi:10.1038/s41598-021-83582-6)
Supplement: Supplementary file 1 — Supplementary Information [file 41598_2021_83582_MOESM1_ESM.pdf]

Machine Learning Enables Prompt Prediction of Hydration Kinetics of Multicomponent Cementitious Systems

Jonathan Lapeyre[1]; Taihao Han[1]; Brooke Wiles[1]; Hongyan Ma[2]; Jie Huang[3]; Gaurav Sant[4]; Aditya Kumar[1*]

1. Department of Materials Science and Engineering; Missouri University of Science and Technology, Rolla, MO.
2. Department of Civil, Architectural and Environmental Engineering; Missouri University of Science and Technology, Rolla, MO.
3. Department of Electrical and Computer Engineering; Missouri University of Science and Technology, Rolla, MO.
4. Department of Civil and Environmental Engineering; University of California, Los Angeles, CA.

*Corresponding author

Aditya Kumar

Assistant Professor, Department of Materials Science and Engineering

Missouri University of Science and Technology, Rolla, MO 65409

Email: kumarad@mst.edu; Phone: 573-341-6994

1.0. Materials and Methods

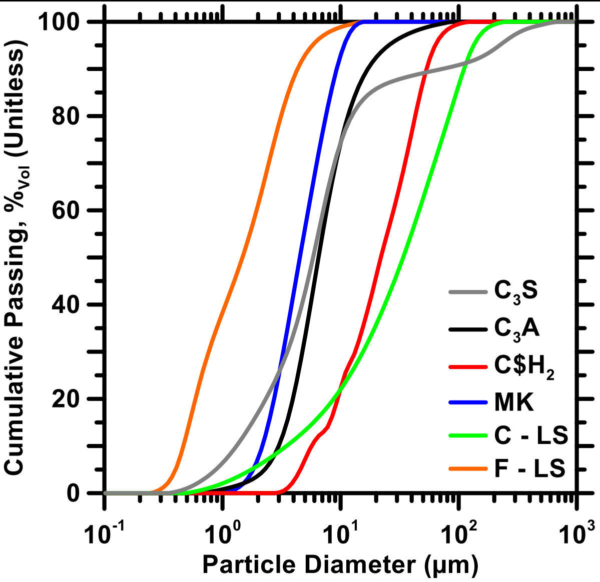

Figure S1: Cumulative PSDs of precursors – tricalcium silicate (C_3S); tricalcium aluminate (C_3A); gypsum (CH_2$) – used for formulation of synthetic cement; and the PSDs of mineral additives: metakaolin (ML); coarse limestone (C-LS); and fine limestone (F-LS).

Table S1: Details of particle size distributions (PSDs) of powders used in this study. For each PSD, the specific surface area (SSA) of the particulates was calculated based on the assumption that the particles have a spherical morphology, while having limited surface roughness and internal porosity. SSAs calculated from this method are expected to be smaller than values experimentally measured from the Brunauer-Emmett-Teller (BET) method by a factor of ~1.6-to-2.2[1–3].

Powder Compound	10th Percentile, (d_{10})	Median Particle size, (d_{50})	90th Percentile, (d_{90})	Specific Surface Area ($m^2 \cdot kg^{-1}$)
C_3S	1.315	5.850	72.47	5760
C_3A	2.961	6.534	17.20	3550
CH_2$	5.680	21.91	53.46	1360
MK	2.175	4.520	9.270	5940
C – LS	3.488	34.03	113.0	2380
F – LS	0.466	1.476	4.330	20800

2.0. Database and Appraisal of Prediction Accuracy of the Random Forests Model

Table S2: Minimum, maximum, mean, and standard deviation of the 9 attributes (7 inputs and 2 outputs) of the training database. The database consists of 5640 unique data-records.

Attribute	Unit	Min.	Max.	Mean	Std. Dev.
C_3S content	$\%_{mass}$	28	100	54.65	15.98
C_3A content	$\%_{mass}$	0	12	4.363	2.990
$C\$H_2$ content	$\%_{mass}$	0	18	4.389	3.851
Metakaolin content	$\%_{mass}$	0	60	15.63	16.58
Limestone content	$\%_{mass}$	0	60	20.95	17.33
Limestone SSA	$cm^2. g^{-1}$	0	20800	9330	9488
Time	hour	1	24	12.50	6.922
Cumulative heat	$J. g_{Cem}^{-1}$	0.4584	375.19	152.7	105.8
Heat flow rate	$mW. g_{Cem}^{-1}$	0.000	13.659	3.244	2.051

Table S3: Minimum, maximum, mean, and standard deviation of the 9 attributes (7 inputs and 2 outputs) of the training database. The database consists of 168 unique data-records.

Attribute	Unit	Min.	Max.	Mean	Std. Dev.
C_3S content	$\%_{mass}$	35.2	79.2	51.88	13.47
C_3A content	$\%_{mass}$	0	8.4	4.314	2.829
$C\$H_2$ content	$\%_{mass}$	0	12.6	4.514	3.833
Metakaolin content	$\%_{mass}$	0	40	14.28	13.86
Limestone content	$\%_{mass}$	0	60	25	15.35
Limestone SSA	$cm^2. g^{-1}$	0	20800	8788	8972
Time	hour	1	24	12.5	6.922
Cumulative heat	$J. g_{Cem}^{-1}$	1.244	361.5	145.8	107.2
Heat flow rate	$mW. g_{Cem}^{-1}$	0.1093	9.231	3.231	2.127

3.0. Prediction of Time-dependent Hydration Kinetics of Cement

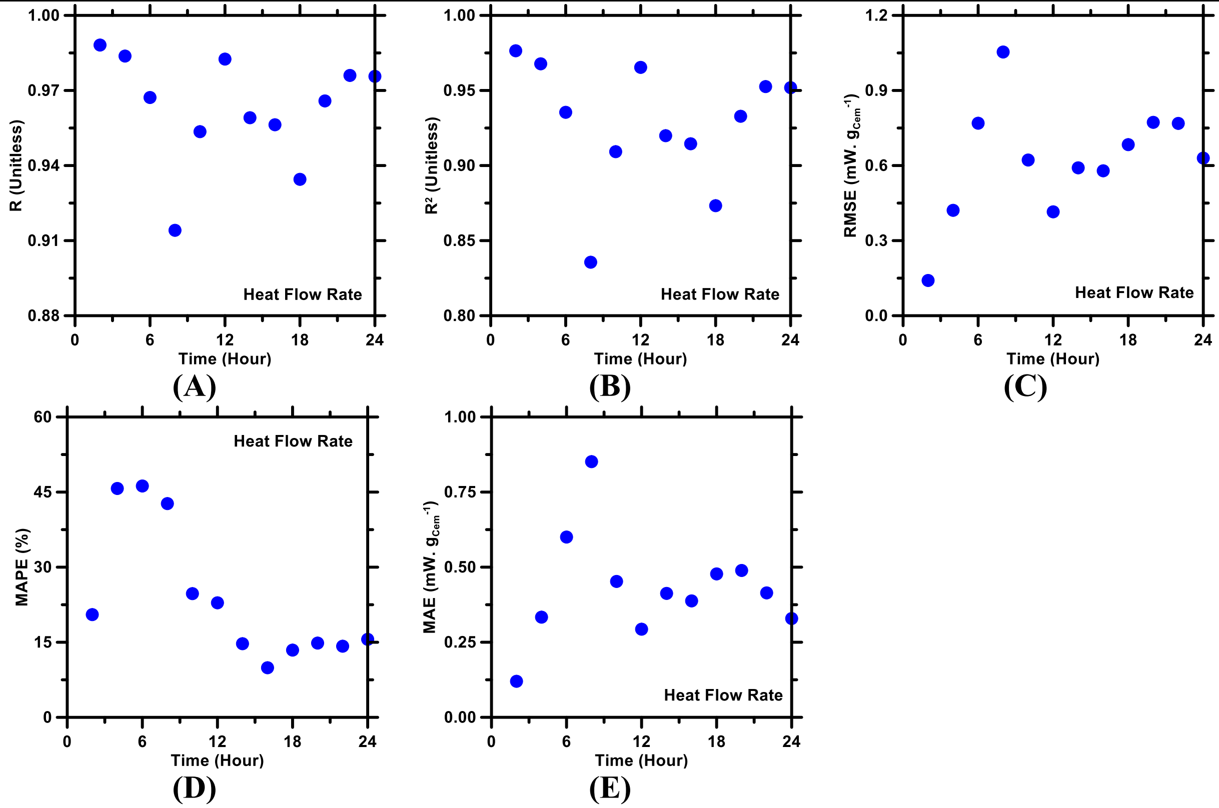

Figure S2: Statistical parameters describing errors in predictions of heat flow rate of pastes over a 24-hour period in the testing database: **(A)** R; **(B)** R^2; **(C)** RMSE; **(D)** MAPE; and **(E)** MAE as functions of time.

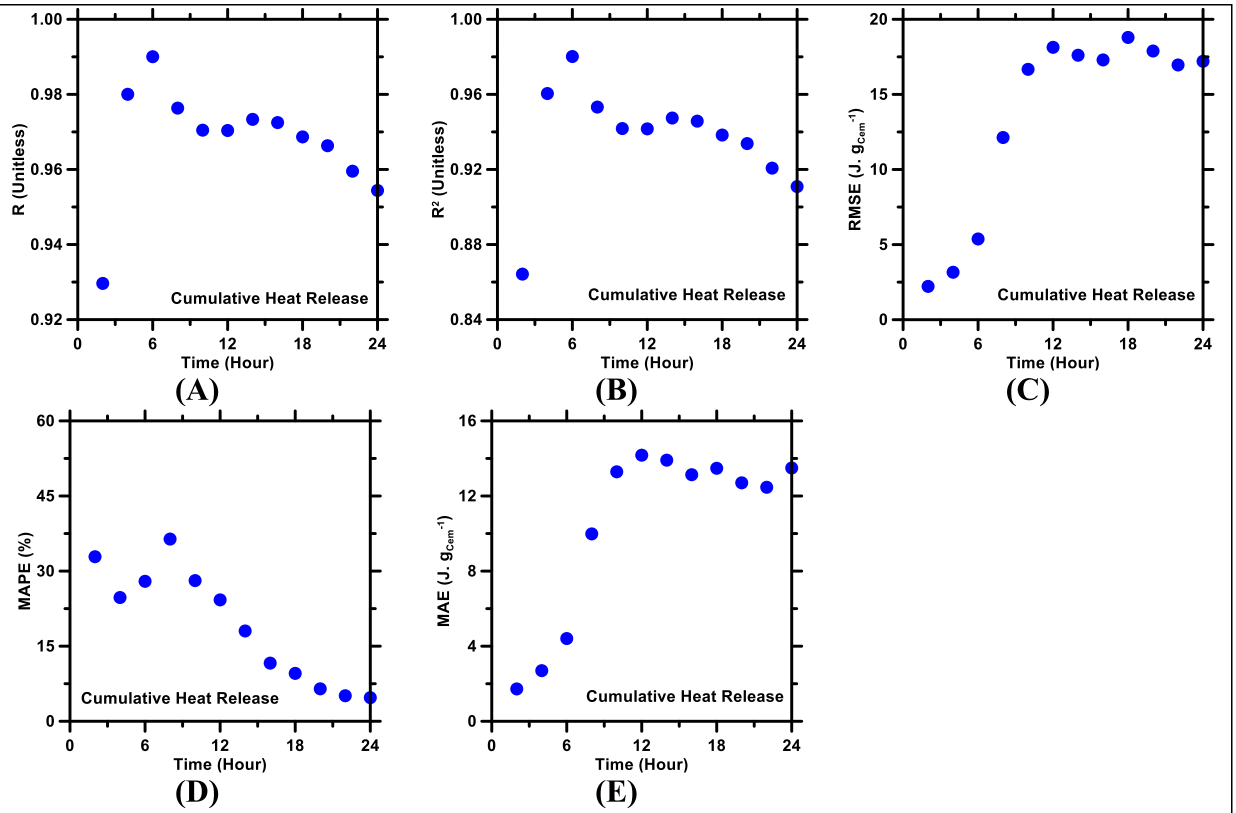

Figure S3: Statistical parameters describing errors in prediction of cumulative heat release of pastes over a 24-hour period in the testing database: **(A)** R; **(B)** R^2; **(C)** RMSE; **(D)** MAPE; and **(E)** MAE as functions of time.

The learning behavior of the RF model – that is, enhancement in prediction accuracy of the model in relation to increasing volume of the training database – is shown in **Figure S4**. To produce training databases of reduced volume (i.e., 90-to-40% of the original volume), data-records pertaining to random pastes were successively removed. Then, the model was trained using the abridged database, and its prediction performance was evaluated on the testing database. As can be seen, the Person correlation coefficient (R) monotonically increases, and the mean absolute percentage error (MAPE) monotonically decreases with increasing volume of the training database. This unambiguously shows that the prediction performance of the ML model improves as it progressively trained with data pertaining to more and more systems. This corroborates an important argument made in the paper: Training the ML model with a database that features greater

diversity (in terms of physicochemical properties of pastes) and greater volume (in terms of number of distinct pastes) would result in improvement of its prediction performance, and therefore its ability to predict optimal mixture designs with high fidelity.

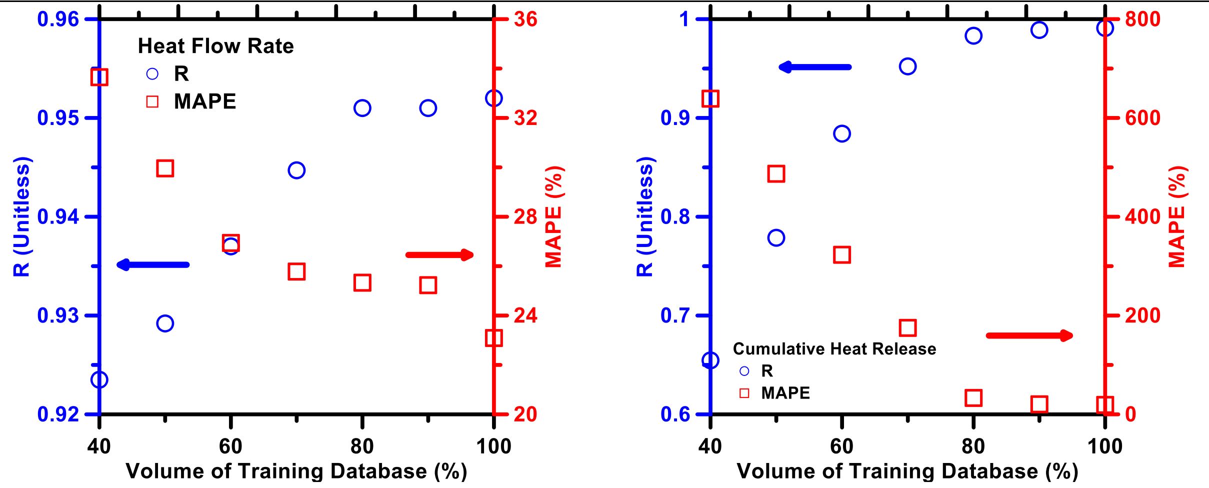

(A) (B)

Figure S4: Enhancement in prediction performance of the RF model – in terms of predicting **(A)** heat flow rate and **(B)** cumulative heat release of pastes in the testing database – in relation increasing volume of the training database. The database's volume was reduced (from its original volume) by randomly selecting pastes, and removing data-records corresponding to them.

Figure S5 ranks the *importance* of each physicochemical attributes in terms of its ability to influence heat flow rate and cumulative heat release. As can be seen, for predictions of both heat flow rate and cumulative heat release, time is the most influential variable. This is expected because with increasing time of hydration, cumulative heat increases monotonically, and the heat flow rate profile transitions through four different stages, that is, initial dissolution period, induction period, acceleration, and deceleration[4,5]. The specific surface area (SSA) of limestone is another crucial factor that affects cement hydration. Previous studies[4,6,7] have shown that a finer filler, owing to its intrinsic filler effect, provides additional sites for nucleation growth of hydration product, thereby accelerating hydration kinetics and affecting heat release behavior. $C\$H_2$ is the third important factor because of its ability to react vigorously with the C_3A phase of cement, while

indirectly affecting the kinetics of hydration of C_3S. For example, when a large amount of sulfate is present in the system, its reaction with C_3A can significantly decelerate the hydration kinetics of C_3S[4,8,9]. All other physicochemical attributes have finite importance, although lower than the attributes described above. From **Figure S5**, it is also clear that all attributes exert greater influence on the heat flow rate compared to cumulative heat release. This is expected because the heat flow rate profile – being the derivative of cumulative heat release – is more sensitive to changes in the material's attributes. The cumulative heat release profile – which always increases monotonically with time – is comparatively less sensitive.

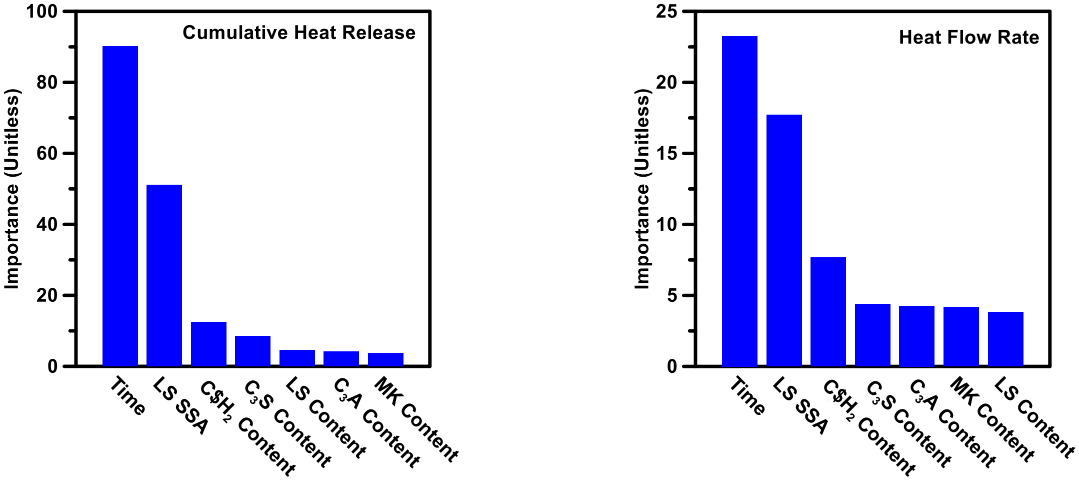

(A) **(B)**

Figure S5: The importance of pastes' physicochemical attributes in terms of their contributions towards: **(A)** cumulative heat release; and **(B)** heat flow rate. Parameters are ranked, from left to right, based on their decreasing influence on the property.

For the purposes of benchmarking the RF model, two additional ML models – developed and used in several of our previous studies[10–14] – were used: support vector machine (SVM); and multilayer perceptron-artificial neural network (MLP-ANN). We consistently found – as we did in our previous studies[10–14] – that the RF model produces more accurate predictions compared to the SVM and MLP-ANN models (see **Figure S6** and **Table S4**). In another study[10], we have

explained various reasons why SVM and MLP-ANN models have inferior prediction accuracy compared to the RF model; especially when the dataset consists of highly nonlinear and nonmonotonic correlations.

Table S4: Prediction performance of ML models (RF; SVM; and MLP-ANN), measured on the basis of the cumulative heat release and heat flow rate of pastes in the testing database. Five statistical parameters (i.e., *R, R^2, MAE, MAPE,* and *RMSE*) are shown.

	ML Model	R	R^2	MAE	MAPE	RMSE
	Unit	*Unitless*	*Unitless*	mW. g_{opc}^{-1}	*%*	mW. g_{opc}^{-1}
**Heat Flow Rate**	MLP-ANN	0.948	0.899	0.507	47.629	0.653
	SVM	0.950	0.903	0.430	27.034	0.651
	RF	0.952	0.912	0.410	23.090	0.631
	Unit	*Unitless*	*Unitless*	J. g_{opc}^{-1}	*%*	J. g_{opc}^{-1}
**Cum. Heat Release**	MLP-ANN	0.988	0.976	13.017	33.470	16.452
	SVM	0.989	0.978	11.337	25.617	15.836
	RF	0.991	0.982	10.453	19.147	14.898

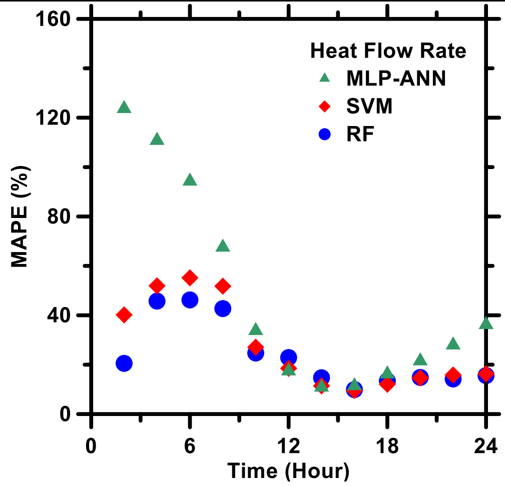

(A)

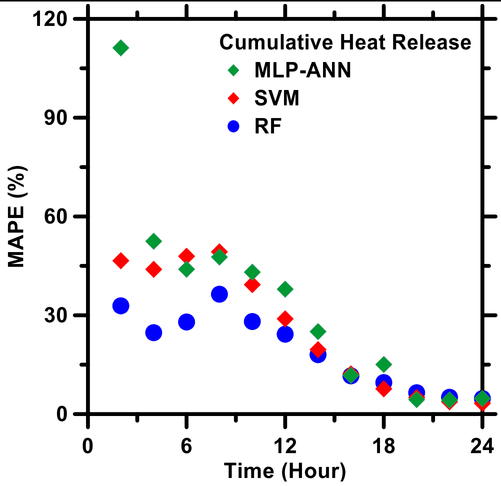

(B)

Figure S6: Comparison of prediction performance of the RF model – in terms of predicting **(A)** heat flow rate and **(B)** cumulative heat release of pastes – against those of SVM and MLP-ANN models. MAPE is used as the representative measure of error here. Other statistical parameters (e.g., R^2) also show that the RF model consistently produces more accurate predictions that the other two models.

References

(1) Lapeyre, J.; Kumar, A. Influence of Pozzolanic Additives on Hydration Mechanisms of Tricalcium Silicate. *J. Am. Ceram. Soc.* **2018**, *101* (8), 3557–3574. https://doi.org/10.1111/jace.15518.

(2) Garboczi, E. J.; Bullard, J. W. Shape Analysis of a Reference Cement. *Cem. Concr. Res.* **2004**, *34* (10), 1933–1937.

(3) Banala, A.; Ma, H.; Kumar, A. Influence of Particulate Geometry on Permeability of Porous Media. *Powder Technol.* **2019**, *345*, 704–716. https://doi.org/10.1016/j.powtec.2019.01.064.

(4) Bullard, J. W.; Jennings, H. M.; Livingston, R. A.; Nonat, A.; Scherer, G. W.; Schweitzer, J. S.; Scrivener, K. L.; Thomas, J. J. Mechanisms of Cement Hydration. *Cem. Concr. Res.* **2011**, *41* (12), 1208–1223. https://doi.org/10.1016/j.cemconres.2010.09.011.

(5) Scherer, G. W.; Zhang, J.; Thomas, J. J. Nucleation and Growth Models for Hydration of Cement. *Cem. Concr. Res.* **2012**, *42* (7), 982–993. https://doi.org/10.1016/j.cemconres.2012.03.019.

(6) Oey, T.; Kumar, A.; Bullard, J. W.; Neithalath, N.; Sant, G. The Filler Effect: The Influence of Filler Content and Surface Area on Cementitious Reaction Rates. *J. Am. Ceram. Soc.* **2013**, *96* (6), 1978–1990. https://doi.org/10.1111/jace.12264.

(7) Puerta-Falla, G.; Kumar, A.; Gomez-Zamorano, L.; Bauchy, M.; Neithalath, N.; Sant, G. The Influence of Filler Type and Surface Area on the Hydration Rates of Calcium Aluminate Cement. *Constr. Build. Mater.* **2015**, *96*, 657–665. https://doi.org/10.1016/j.conbuildmat.2015.08.094.

(8) Marchon, D.; Flatt, R. J. 8 - Mechanisms of Cement Hydration. In *Science and Technology of Concrete Admixtures*; Aïtcin, P.-C., Flatt, R. J., Eds.; Woodhead Publishing, 2016; pp 129–145. https://doi.org/10.1016/B978-0-08-100693-1.00008-4.

(9) Quennoz, A.; Scrivener, K. L. Interactions between Alite and C3A-Gypsum Hydrations in Model Cements. *Cem. Concr. Res.* **2013**, *44*, 46–54.

(10) Cook, R.; Lapeyre, J.; Ma, H.; Kumar, A. Prediction of Compressive Strength of Concrete: A Critical Comparison of Performance of a Hybrid Machine Learning Model with Standalone Models. *ASCE J. Mater. Civ. Eng.* **2019**, *31* (11), 04019255. https://doi.org/10.1061/(ASCE)MT.1943-5533.0002902.

(11) Han, T.; Stone-Weiss, N.; Huang, J.; Goel, A.; Kumar, A. Machine Learning as a Tool to Design Glasses with Controlled Dissolution for Application in Healthcare Industry. *Acta Biomater.* **2020**, *107*, 286–298. https://doi.org/10.1016/j.actbio.2020.02.037.

(12) Han, T.; Siddique, A.; Khayat, K.; Huang, J.; Kumar, A. An Ensemble Machine Learning Approach for Prediction and Optimization of Modulus of Elasticity of Recycled Aggregate Concrete. *Constr. Build. Mater.* **2020**, *244*, 118271. https://doi.org/10.1016/j.conbuildmat.2020.118271.

(13) Cook, R.; Keitumetse, C. M.; Hayat, M. B.; Kumar, A.; Alagha, L. Prediction of Flotation Performance of Sulfide Minerals Using an Original Hybrid Machine Learning Model. *Eng. Rep.* **2020**, *12* (NA), e12167. https://doi.org/10.1002/eng2.12167.

(14) Zhuang, Y.; Yang, Q.; Han, T.; O'Malley, R.; Kumar, A.; Gerald II, R.; Huang, J. Fiber Optic Sensor Embedded Smart Helmet for Real-Time Impact Sensing and Analysis through Machine Learning. *J. Neurosci. Methods* **2021**, *351* (NA), 109073. https://doi.org/10.1016/j.jneumeth.2021.109073.
